# Regeneration, Regengrow and Tissue Repair in Animals: Evolution Indicates That No Regeneration Occurs in Terrestrial Environments but Only Recovery Healing

**DOI:** 10.3390/jdb13010002

**Published:** 2024-12-30

**Authors:** Lorenzo Alibardi

**Affiliations:** Comparative Histolab Padova, 35100 Padova, Italy; lorenzo.alibardi@unibo.it

**Keywords:** metazoans, regeneration, healing, development, evolution

## Abstract

The present, brief review paper summarizes previous studies on a new interpretation of the presence and absence of regeneration in invertebrates and vertebrates. Broad regeneration is considered exclusive of aquatic or amphibious animals with larval stages and metamorphosis, where also a patterning process is activated for whole-body regeneration or for epimorphosis. In contrast, terrestrial invertebrates and vertebrates can only repair injury or the loss of body parts through a variable “recovery healing” of tissues, regengrow or scarring. This loss of regeneration likely derives from the change in genomes during land adaptation, which included the elimination of larval stages and intense metamorphosis. The terrestrial conditions are incompatible with the formation of embryonic organs that are necessary for broad regeneration. In fact, no embryonic organ can survive desiccation, intense UV or ROS exposition on land, and rapid reparative processes without embryonic patterning, such as recovery healing and scarring, have replaced broad regeneration in terrestrial species. The loss of regeneration in land animals likely depends on the alteration of developmental gene pathways sustaining regeneration that occurred in progenitor marine animals. Terrestrial larval stages, like those present in insects among arthropods, only metamorphose using small body regions indicated as imaginal disks, a terrestrial adaptation, not from a large restructuring process like in aquatic-related animals. These invertebrates can reform body appendages only during molting, a process indicated as regengrow, not regeneration. Most amniotes only repair injuries through scarring or a variable recovery healing, occasionally through regengrow, the contemporaneous healing in conjunction with somatic growth, forming sometimes new heteromorphic organs.

## 1. Developmental Biologists Need Zoological Information to Grasp Regeneration

**Figure 1:**
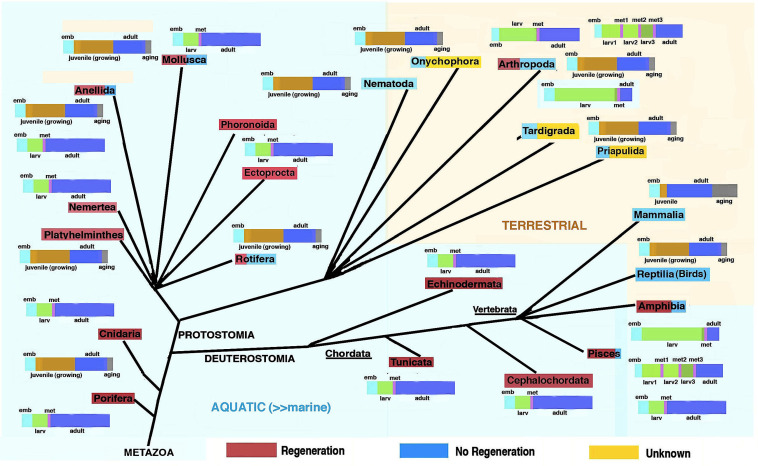
Summarizing image reporting the distribution of regenerative abilities among extant aquatic (light-blue background) and terrestrial (pale-brown background) animals. The colored bars associated with the different animal phyla represent the progressive phases of their main life cycles (embryo phase in pale blue, larval phase in green, metamorphic phase in vermillion, juvenile and growing phase in brown, adult phase in deep blue, and aging phase in grey). Phyla including metamorphosis (vermillion color segment inside their life cycle bars) also show large regenerative ability and are generally marine. Among arthropods, one or more metamorphic transitions occur in insects, but these are mainly body growths through molting in heterometabolous insects and large changes derived from localized imaginal disks in holometabolous insects (see text for further explanation). In minor phyla (Tardigrada, Onychophora, Priapulida but also Phoronida, Ectoprocta and Rotifera), no or insufficient information on regeneration is available.

Repair and regeneration are basic life properties, but their activation varies, from cell, tissue, organ and, finally, whole body regeneration [1,2,3,4,5,6,7,8,9,10,11,12,13]. Before dealing with the regeneration process present in different invertebrates and vertebrates (Figure 1), a brief consideration on the biological status of these extant animals should be made. Aside from the specific life cycles of different animals, the presence, amount and distribution of adult stem cells (ASCs) in different invertebrates and vertebrates should also be considered for explaining animal regeneration [14]. The present, brief manuscript reports and extends previous theoretical studies that have considered the process of “evolution” as the unifying vision aiming to explain animal regeneration in biological terms [15,16,17,18,19]. In order to introduce the current thoughts about this evolutionary hypothesis, some of the previous concepts have to be repeated here.

**Figure 2:**
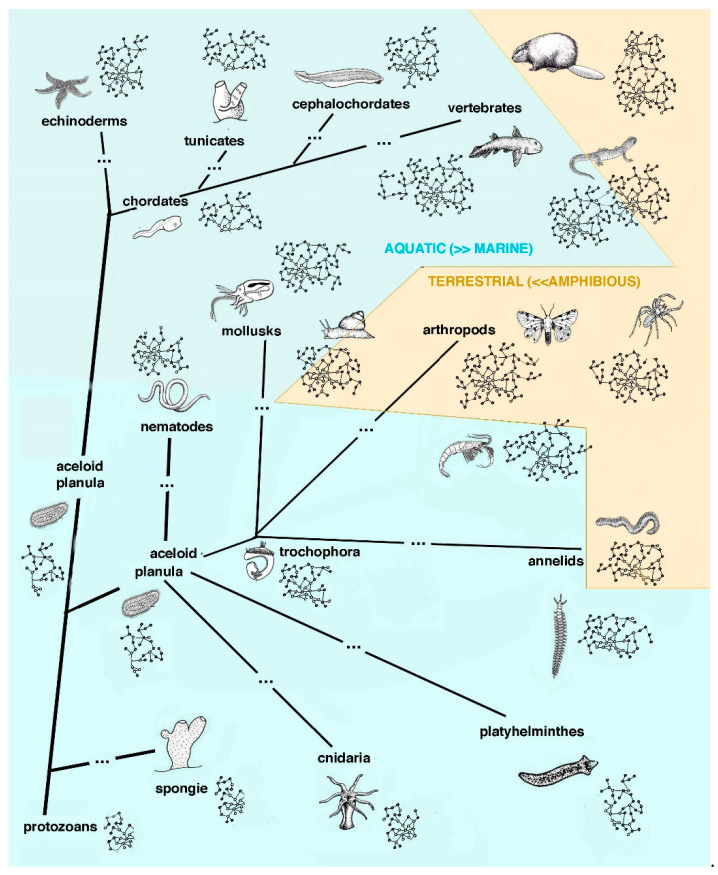
Schematic drawing showing the indicative evolution of the main phyla of animals, from a primitive planula and/or aceloid progenitor in the marine environment (light-blue background) to a marine or a terrestrial environment (light-brown background). Dots along the evolutionary lines indicate elapsed time (millions of years) to evolve new animals. Each representative animal form is associated with an idealized simple genome network of different extension, according to the animal complexity (small in simple invertebrates and larger in more complex marine or terrestrial invertebrates and vertebrates). The final genome network of extant animals is invariant and includes developmental genes (or groups of genes), some of which can be re-activated in adult animals after injury, promoting regeneration. All terrestrial animals (light-brown background) lost a larval phase or evolved a terrestrial adapted larva (insects) and also lost metamorphosis and broad regenerative ability. Terrestrial restrictions imposed the evolution of a rapid healing process, generally through scarring.

**Figure 3:**
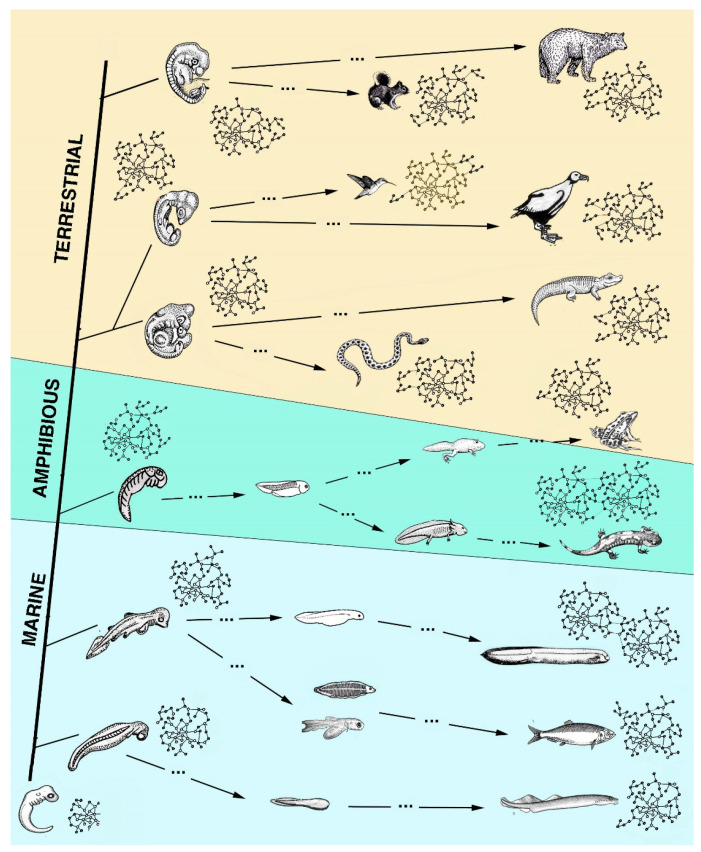
Schematic drawing for vertebrate evolution in relation to regeneration. From a basic embryonic form at the base of the tree, different embryos were derived and gave rise to cyclostomes and different types of fish (light-blue background), then amphibians (green background), and finally terrestrial vertebrates, reptiles, birds and mammals (pale-brown background). Each embryo or adult form is associated with an idealized gene network of different complexity. Dots along the evolutionary lines indicate elapsed time (millions of years) to evolve new animals. Numerous fish and amphibians possess one or more larval phases with one or more metamorphic transitions and, after an injury as adults, can often broadly regenerate. This is not the case for amniotes (terrestrial vertebrates) that lost a larval form and related metamorphosis during land adaptation and also do not regenerate after injury or have limited healing recovery, regengrow or heteromorphic regeneration in rare cases and generally scar.

Each extant species has followed a specific evolution since the pre-Cambrian era and during the Paleozoic era and has evolved a specific life cycle, from the embryonic stage to the adult, the final stage of the cycle (see colored bars in Figure 1). In the Paleozoic era, from about 570 million years ago (Cambrian) to the Cenozoic era, and in the last 1–2 million years, different groups of invertebrates and vertebrates have evolved in environments present on the land. The new environments presented radical differences with their original marine environment [20]. During this evolution, animal genomes changed in relation to the environment where these animals were adapting and presently live in (Figure 2 and Figure 3).

It has been hypothesized that epigenetic modifications took place during this evolution [16,19]. These changes have produced the anatomical, physiological and biochemical characteristics typical for each extant species, sponges, cnidaria, platyhelminthes, nematodes, annelids, mollusks, arthropods and different chordates. Among these characteristics is also included the ability to recover after injuries through a variable cell proliferation and growth of tissues, organs or even the entire body, the latter a basic capacity of various marine animals [4,15,16]. Originally born in the seas, numerous invertebrates (protostomes and deuterostomes) remained in the marine environments until present time, evolving specific adaptations to different ecological niches but largely maintaining the original regenerative ability. Many of these organisms contain numerous ASCs (adult stem cells) that can be activated for regeneration (for extensive details, see [14]). In comparison, invertebrates and vertebrates that became terrestrial had to evolve specific characteristics that suited them to the new conditions present on land, mainly the scarcity of water, increased UV-radiation, the presence of high levels of oxygen with the production of damaging ROS (reactive oxygen species), high microbial exposure, mechanical resistance to land gravity, ability to move and interact on a solid substrate, new form for offspring dispersal, etc. These new requirements necessitated changes in some critical characteristics within the original life cycle of each species, including some that also determined lowering and even the compete loss of the original regenerative ability [17,18,21].

Therefore, what we observe today about the distribution of the regenerative capacity among animals (Figure 1) reflects these evolutionary modifications of the genomes that took place during millions of years, a unique and invariant process. In fact, once a specific evolution took place in different species, erasing regeneration, this was a definitive step that cannot be reversed, re-introducing regeneration, without influencing the remaining anatomical, physiological and biochemical characteristics of those species. However, in case some form of cellular recovery or tissue regeneration re-appeared in an evolutionary lineage that primarily lost the ability to regenerate, the renovated ability to regenerate is, however, limited since evolution cannot be or minimally reversed.

In conclusion, any study on the regeneration of extant animals is primarily concerned with the understanding of the specific regenerative process of those animals; therefore, it is research on post-embryonic developmental stages. Consequently, the information on regeneration processes derived from the study of classical models of regeneration such as planarians, hydras, starfish, sea squirts and amphibians can only explain regeneration in those species. The information gained from these invertebrate and vertebrate models cannot be easily translated into attempts to promote regeneration in other species, particularly the non-regenerating ones, with the risk to modify the characteristics reached in different species during their specific evolution [15,16,17,18]. Despite this biological reality, some terrestrial-adapted species have evolved various degrees of reparative abilities, often referred to as “regeneration” (appendage regeneration in arthropods, heteromorphic limb regeneration in some amphibians, tissue physiological regeneration, liver regeneration in vertebrates, digit tip regeneration in rodents, heteromorphic tail regeneration in lizards, horn or antler regeneration in cervids, etc.). As it will be indicated next, these sometime remarkable recovering abilities, likely derived from the evolution of alternative development pathways, which details remain to be discovered, are permissive within the limits imposed from terrestrial life (mainly, dryness, UV and high oxygen levels), and there is also the presence of adult body cells surrounding and invading the regenerating mass of cells, which interactions and immunological competition may interfere negatively with regeneration.

## 2. Regeneration, Regengrow, Recovering Healing and Scarring

From the simple aquatic animals, invertebrates, such as sponges, cnidaria and platyhelmintes, to the more complex aquatic and terrestrial invertebrates and vertebrates, such as mollusks, arthropods, echinoderms and chordates, have variable healing abilities present, generally referred to as “regeneration”. In the present paper, however, the term regeneration is only applied to a post-embryonic morphogenetic process where not only cell proliferation and growth but also patterning with the formation of a morphogenetic gradient is reactivated and restitute a largely complete organ by epimorphosis or even the entire animal (whole body regeneration or morphallaxis). Other repairing abilities among animals, often termed regeneration, are here indicated as variable “recovering healing” such as tissue regeneration, “heteromorphic regeneration” (the restitution of mixed tissues forming an organ similar but simplified with respect to the lost original organ), “regengrow” (healing associated with somatic growth), and “scarring” (the replacement of functional tissues with irregular dense connective tissue). Therefore, the healing of relatively small organs or tissues (digit tips, ear holes, skin areas, etc.) or rare examples of larger organs made by a prevalent tissue (epithelial intestine, hair or feather replacement, liver, cervid horns, etc.) are here consider as “recovering healing” or as “cyclical physiological tissue regeneration” [1,22].

The accumulation of data and papers on animal regeneration, particularly planarians, hydras and amphibians, are overwhelming and eventually essential to grasp the mechanisms of broad regenerations. It is rational stating that the different wound healing and regenerative abilities among animals eventually rest upon the differential but specific gene activation present in each taxonomic group, invertebrates or vertebrates [2,13,23,24,25,26,27,28,29,30,31]. However, this information by itself does not allow understanding why this epiphenomenon is present only in some extant species, a process that can only be explained considering the evolution of these different animals in relation to their environment.

Most studies dealing with regeneration present a list of genes or proteins activated or de-activated during the process, basically catalogues of genes turned on or off and genes lost, modified or changed. For instance, the genes *Ras-dva1* and *Ras-dval-2*, sustaining regeneration in anamniotes, have been lost in amniotes that cannot regenerate [7,13,32,33]. Also, the introduction of the human gene *arf-tumor suppressor* in the zebrafish determines the loss of tail regeneration in this fish [34]. The knowledge of the molecular details by itself is, however, insufficient to grasp the process of animal regeneration that needs the knowledge of the ecological adaptations and evolution of different animals in order to reach a biological synthesis and understanding on why some animals can or cannot regenerate. Little general and synthetic interpretation from these molecular studies about regeneration has come out in past years, but a few recent papers have started to address the problems in molecular and evolutionary terms for vertebrates [7,13,33,35]. In the latter studies, a list of genes, directly or indirectly involved in regeneration that have been lost from anamniotes to amniotes, is reported, indicating critical biomolecular and structural novelties present in amphibious versus terrestrial vertebrates.

The only way to address the understanding of “regeneration” in a biological context must consider the specific evolution of different animals. Therefore, in new attempts to grasp the sense of regeneration, the zoological knowledge of different animal models of regeneration and the evolutionary explanation of their present body organization are needed to address molecular studies. Thinking in terms of evolutionary changes can supply an intellectual framework on which one can re-ordinate the long list of cell processes and genes activated during regeneration in invertebrates and vertebrates [2,10,13,23,24,25,26,27,28,29,30,31].

Within this idea, the following points should be considered: (1) while focusing on regeneration, the specific evolutionary status or environment of different animals should be considered; (2) regeneration is a post-embryonic epiphenomenon; therefore, it can only partially repeat development in any species, from high (planarians, hydras, ribbon worms, starfish, tunicates, etc.) to low or no (arthropods and amniotes) regeneration; (3) regeneration has specifically evolved in different species of invertebrates and vertebrates together with other specific functional and morphological characteristics that are not separable from regeneration; (4) regeneration in adult animals utilizes many, some or few of the developmental gene pathways for the morphogenesis of different organs that were utilized during embryogenesis; (5) the transcriptomes of organ regeneration in different animals should be compared with the transcriptomes of the same organs during development. This comparison can reveal “alternative developmental gene pathways” utilized in different animals for regenerating injured organs, some of which were previously utilized during the embryonic development of those organs.

## 3. Growth Rates and Telomerase Influence Healing Abilities and Regeneration

Different species grow with specific patterns, so regeneration studies have to consider the presence of growth during repair or regeneration in different animals [36]. Comparative studies on invertebrates and vertebrates have detected a different distribution of “adult stem cells” (ASCs) within these animals, cells that are also utilized for organ regeneration after injury [14]. ASCs last long or even a lifetime inside the body and are frequent in invertebrates, especially marine, but scanty and with lower pluri- or multi-potentiality in most vertebrates. Most growing animals contain regions where cell proliferation is active and continuously contributes to the volumetric increase in organs and of the entire body, or their physiological regeneration or cell turnover (epidermis, epithelial intestine, blood, bone epiphyses and their growth plates, etc.). When an injury affecting organs or a larger amputation takes place in a growing animal, the healing process varies in timing, from days to months, but the injured organ also continues to grow during this period until the full somatic growth of that species is reached. When “recovery healing”, often indicated as regeneration, is contemporaneous with physiological growth, the cells produced from growth and those stimulated to regenerate after injury are not distinguishable. Therefore, almost all processes indicated as “regeneration” in post-embryonic stages of growing animals, invertebrates or vertebrates derive from a variable contribution of proliferating cells for somatic growth with those for healing (regeneration), a process indicated as *regengrow* [15,16,17,21]. This recovering healing does not activate a patterning process that is instead a typical embryonic characteristic that is instead re-activated during a broad process of organ regeneration.

**Figure 4:**
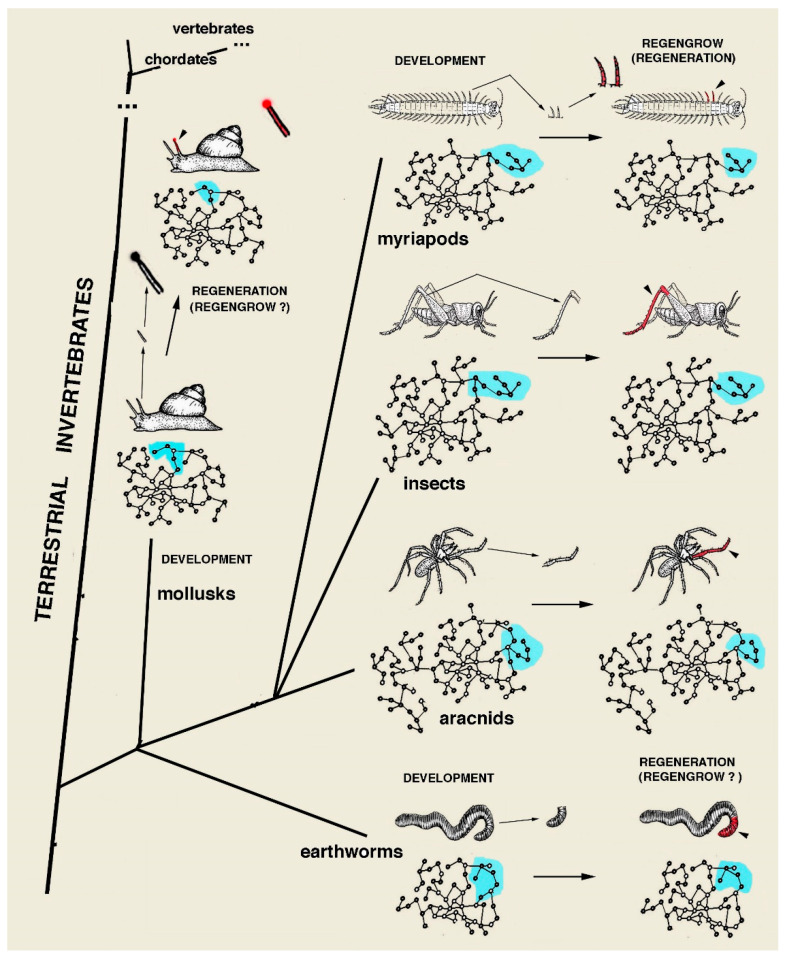
Schematic drawing illustrating few examples of *regengrow* or of recovery healing after loss (small arrows) of appendages in terrestrial animals (myriapods, insects and arachnids), eyed-peduncles in the head (gastropod mollusks), or posterior sections of the body (earthworms). Within the associated gene networks for each animal, in light-blue colored areas, are indicated the idealized region of the genome utilized for the development of the specific appendages (left) or regions of the body that are later lost (small arrows) and regenerated (large arrows and genomes on the right). Regenerated organs are colored in red. Note that in these simple examples, the light-blue areas of the genome utilized for regeneration (on the right) are smaller than those utilized for the development of the same organs (on the left). This ideally indicates that not all developmental genes can be re-activated for regenerating the lost organs, but they are, however, sufficient to restitute all or most of the lost organ (see text and Alibardi, 2023 a–c, for more explanation).

**Figure 5:**
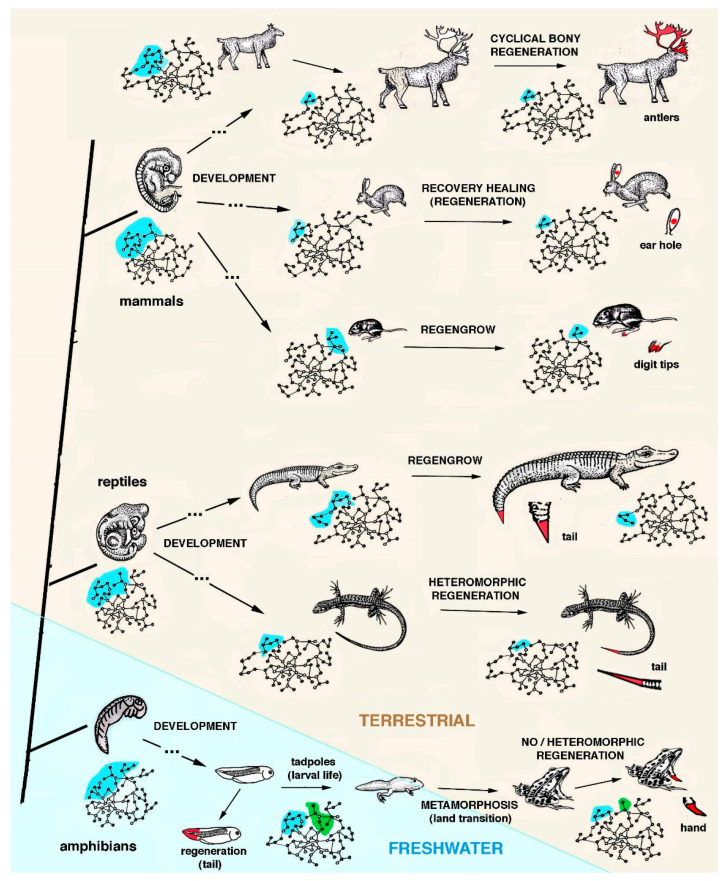
Schematic drawing featuring a few examples of different healing abilities among amphibians (light-blue background) and amniotes (light-brown background). The light-blue areas within the gene networks for each embryo or adult animals ideally represent genes utilized for development (on the left) and for recovery healing after injury or loss of the same organs (arrows on the right). The regenerated organs are colored in red. Ample regeneration is present in freshwater anuran tadpoles, but regeneration is lost at metamorphosis, and, rarely, heteromorphic appendages (arms or digits) are regenerated/regengrown. The light-blue area present in the gene network associated to tadpoles ideally indicates genes utilized during development that, in combination with genes utilized for metamorphosis, also determine regeneration. Heteromorphic but large regeneration occurs in lizard tail and, occasionally, in growing crocodilians (*regengrow*). In mammals, aside from the prevalent scarring outcome and physiological tissue regeneration, three examples of “recovery healing” are shown: cyclical bony regeneration in deer or reindeers after drop of antlers in previous cycles, ear hole recovering in hares, and digit tips *regengrow* in rodents (see text for further explanation). Within the gene networks associated with each animal, the light-blue areas ideally indicate those sections of the genome utilized for developing the specific organ or appendage (on the left) and those re-activated for healing or regenerate the same organ (arrows on the right). Note the smaller light-blue areas utilized for healing or regeneration in each idealized gene network, in comparison to those utilized for development (on the left), an indication of limited re-activation of the same or closely related genes.

Among land species, *regengrow* associated with molting occurs for limb-antennae recovering (indicated as regeneration) in crustaceans, insects, myriapods and arachnids (Figure 4). In vertebrates, *regengrow* or recovery healing occurs during skin or ear healing in some rodents or lagomorphs, in digit tip recovering (indicated as regeneration) and in rare cases of tail tip regeneration in crocodilians (Figure 5). In the best cases of “recovery healing”, named heteromorphic regeneration, this is observed in some anuran and lizard limbs and in regenerating tails of numerous lizards [35,37,38]. A variable healing repair takes place in few tissues of most mammals that recover small masses of tissues with no patterning [36,39]. This process cannot be repeated more than once and generally derives from injuries that occur in juveniles, as in adults no healing but instead scarring takes place. Marine invertebrates, fish and amphibians, can instead regenerate multiple times, re-patterning their lost organs with a similar but not perfectly identical version of the same processes of development. The best processes of extensive recovery healing among mammals are, however, present in a few species of rodents and lagomorphs (Figure 5). However, this process should not be confused with regeneration as defined in the present discussion, namely, a large organ patterning or complete body restitution in the anatomy and physiology of the injured or lost part. The case of rodents of the genus *Acomys* is the best example of mammalian heteromorphic regeneration [36,39]. Although some amniotes may utilize alternative gene networks, like that proposed during the regeneration of lizard tail [35], neither lizards nor rodents can re-pattern a regenerating organ as in amphibians or in numerous invertebrates [3,6,8,9]. This is an unlikely or even impossible process given the limitations of the terrestrial environment previously indicated.

During embryogenesis, cell multiplication is essential, like telomerases that are activated in transit amplification cells for the continuous production of new cells, and tissues, and the growth of the embryo. Since embryonic development takes place in an aqueous or humid environment (in both aquatic and terrestrial animals), telomerase activity occurs in such hydration status, a primitive condition of life. Mechanisms that control cell proliferation determine a regulation of the telomerase activity, and eventually cells initiate differentiation, limiting telomerase activation. In marine animals, telomerases remain active but are regulated in adult bodies that, like in their embryos, continue to live in a water environment. This is UV-protected, with a relatively low virus and microbe contamination, and low in mutagen pollution in natural conditions [40,41,42].

During animal evolution for land colonization, the environmental conditions changed: dry impeded to hydrate injured bodies in order to form regenerative blastemas (cells dry out), UV exposure to cells increased in sub-aerial conditions (damaging cells), viruses and bacterial infections (especially airborne) and also pollutants could attach the skin and enter the oral–anal cavities. The latter, therefore, necessitate a stronger immune reaction and barrier formation. The new conditions determined a progressive increase in the complexity of the immune system that added to innate immunity responses those of the adaptive immunity. Because of the above processes and the high stimulation of cell replication, it has been hypothesized that telomerase activities were inhibited to avoid tumor activation in the somatic organs of most terrestrial animals [41,42]. Telomerase activity is also contrasted by the high number of ROS produced on the terrestrial environment. The adaptive immune system, aside from protecting from infections, could also detect cancer and regenerating cells that express embryonic/fetal-like antigens, mimicking potentially oncogenic antigens. This ability determined not only the rejection of early-produced tumor-like antigens but also of any cells that expose similar fetal–embryonic antigens such as those needed for regeneration [35,43].

## 4. Conclusions and Perspectives

In conclusion, extant animals derived from specific evolutionary histories that also included their regenerative ability were explored. In particular, in long periods during their evolution to terrestriality, different invertebrates and vertebrates generated invariant genomes, producing extant terrestrial animals well adapted to sub-aerial life but unable to regenerate. This loss was likely derived from epigenetic changes that occurred during land adaptation, but specific genes involved in the process still have to be identified [19].

Future comparative genomic analyses of activated gene programs for development and regeneration are necessary to understand why and how different animals possess broad, intermediate or no regenerative abilities. The hypothesis summarized in this brief manuscript, if confirmed from new modern research studies, could indicate whether some (minor) genetic manipulations will be feasible to improve human healing abilities without any harmful consequences for the individual. The modification of species-specific gene networks that evolved in different animals during millions of years, through man-made genetic manipulations, is not really feasible [15]. According to this reasoning, what can regenerative medicine really expect to achieve in the next decades? Aside from the local genetic manipulation of transferred, genetically engineer cells in damaged organs, or using stem cells [44,45], a bioengineering approach should combine sophisticated anatomical devices with artificial organs originated in bioreactors [46]. For instance, an arm or a leg of the right size, personalized for each patient, could be produced as prosthesis with the appropriate anatomical aspect. The connection of this arm device with the stump nerves of the patient could be achieved surgically in order to gain neuromuscular control of the prosthesis and most of the original function [47]. Also, for inner organs (liver, kidneys, hearts, etc.), attempts in this direction will be feasible, giving rise to bionic patients.

## Data Availability

No other data are available.
